# A psychotic dilemma: psychodynamic group therapy for patients with schizophrenia in times of crisis

**DOI:** 10.3389/fpsyg.2026.1704951

**Published:** 2026-02-26

**Authors:** Mitra Onsori, Meredith Stone, Imke Grimm, Dorothea von Haebler

**Affiliations:** 1 Department of Psychiatry and Psychotherapy, Charité – Universitätsmedizin Berlin, Berlin, Germany; 2 Head Perinatal Psychiatry and Women’s Mental Health, Royal Hospital for Women, Randwick, NSW, Australia; 3 International Psychoanalytical University Berlin, Berlin, Germany

**Keywords:** COVID-19, group psychotherapy, psychodynamic, psychosis, qualitative content analysis, schizophrenia

## Abstract

**Introduction:**

Psychodynamic group therapy (PDGT) for schizophrenia offers a therapeutic space where patients can explore conflicts considered pathognomonic for the disorder. This study examines the role of PDGT during a crisis, focusing on psychotic dilemmas as described by Mentzos while also exploring the relevance of psychodynamic psychosis theory in ensuring effective psychotherapeutic care.

**Materials and methods:**

Using qualitative content analysis, we analyzed transcribed audio recordings of therapy sessions during the COVID-19 pandemic. Thematic categories were inductively developed and examined within Mentzos’ psychodynamic framework.

**Results:**

Four key themes emerged: (1) Loss of something meaningful, reflecting existential fears intensified by the crisis; (2) Paranoia, manifesting as distrust toward external structures; (3) Care and self-care, illustrating oscillations between autonomy and dependence; and (4) Closeness and distance in relationships, highlighting struggles of relationship formation, especially in times of rigid social boundaries. These themes mirror the psychotic dilemmas identified by Mentzos, reinforcing their relevance in crisis settings. The findings suggest that PDGT enables patients to explore these dilemmas and negotiate interpersonal conflicts. However, the crisis environment led to therapy disruptions, impacting group cohesion and participation, likely exacerbating symptoms in some patients.

**Conclusion:**

Psychodynamic group therapy is crucial in supporting patients with schizophrenia, especially during crises, by enabling engagement with core conflicts through relationships rather than symptom formation. However, maintaining therapeutic continuity remains a challenge. Future research should examine group cohesion in various crises and its link to delusional symptoms, as well as strategies to stabilize group therapy and expand PDGT access internationally.

## 1 Introduction

Psychodynamic group psychotherapy (PDGT) for people with schizophrenia and schizoaffective disorders has been practiced for over a century but remains under-researched (Orfanos et al., 2015). Its theoretical foundations rely on the psychodynamic model of schizophrenia pathogenesis developed by theorists such as Bion (1962), as cited by Lempa et al. (2017) or Winnicott (1951), as cited by von Haebler et al. (2022) and the Greek-German psychiatrist and psychoanalyst Mentzos (1997).

Mentzos’ theory describes psychosis as an attempt to resolve “an existentially threatening dilemma” involving the regulation of proximity and distance (von Haebler, 2015b, p. 49). Importantly, psychotic symptoms are a response to inherently contradictory forces, not just a symptom of disease, but also a sign of restorative efforts (akin to Freud’s notion of psychosis as an attempt at restitution) (Freud, 2018; McGlashan, 2009).

Psychodynamic group therapy addresses this dilemma by adapting psychodynamic individual therapy to the group setting (von Haebler et al., 2022). It fosters “intensive empathy” while maintaining “respectful distance” [Mentzos, 2015, p. 232, cited by Lempa et al., 2017, p. 88], thereby enabling dynamic interactions within a structured therapeutic environment. Additionally, PDGT provides a retreat space that helps reduce the intensity of transferential and countertransferential forces in dyadic therapy (Štrkalj Ivezić and Urlić, 2015).

Research suggests that PDGT can be as effective, or even more effective than individual psychodynamic psychosis therapy [Purvis and Mismikis, 1970; O’Brien et al., 1972, as cited by de Chavez (2009)]. The therapeutic alliance among group members and between the group and the therapist is a key factor in this effectiveness, which is crucial in fostering engagement and promoting therapeutic change (Bourke et al., 2021).

Structured interventions within PDGT help address core dilemmas specific to schizophrenia, as emphasized in the Manual for Psychodynamic Psychotherapy of Schizophrenia (Lempa et al., 2017). These structured approaches ensure that group therapy provides a balance between emotional containment and dynamic interpersonal exchange, which has been found essential for maintaining long-term therapeutic engagement (Hartwich, 2015). Beyond engagement, psychoanalytic group settings offer unique benefits in managing psychotic dilemmas, as highlighted by Urlić and de Chavez (2018). The shared therapeutic space allows for the externalization of internal dilemmas, enabling patients to work through their struggles in interaction with others.

Therapeutic work in PDGT primarily aims to protect both the therapeutic frame and all group members, thereby fostering a stance characterized by openness, curiosity, authenticity, emotional firmness, and self-care (Lempa et al., 2017). Interpretative interventions are used sparingly and with low density, comparable to individual psychodynamic therapy, as an excessive attribution of meaning may not be helpful in this context. Instead, therapists focus on holding and containing emerging thoughts and affects, allowing group members to articulate perspectives, limits, and meaning-making processes themselves. The therapeutic stance emphasizes the coexistence of multiple perspectives without premature correction, supporting a respectful exploration of differing views within the group. In line with PDGT manuals, psychotic or idiosyncratic material is therefore not addressed in terms of factual accuracy but explored with regard to its affective and relational significance, a process that is facilitated by the group and often more readily accepted through peer interaction than through direct therapeutic confrontation.

Empirical findings further substantiate these theoretical considerations: Pec et al. (2018) demonstrate that PDGT led to significant improvements in clinical symptoms and quality of life among patients with schizophrenia spectrum disorders, with benefits persisting both at treatment completion and follow-up.

Despite theoretical advancements, clinical research on PDGT remains limited. Addressing this gap is essential to improving psychotherapeutic care, further exploring and demonstrating the benefits of PDGT and gaining a more nuanced understanding of its scope and limitations.

Although PDGT and other psychotherapeutic approaches for psychosis are practiced and researched, psychotherapeutic services for schizophrenia remain underused in Germany and internationally, compared to other diagnostic groups (von Haebler, 2015b; Schlier et al., 2017). This gap in care is particularly concerning, as national and international treatment guidelines recommend psychotherapy for schizophrenia at all stages and severity levels. In the UK, the National Collaborating Centre for Mental Health (UK) (2009) outlines psychotherapy as an essential component of care, while German DGPPN - Deutsche Gesellschaft Für Psychiatrie und Psychotherapie [DGPPN] (2019) similarly advocates its use. The persistent underutilization of psychotherapeutic interventions represents a deviation from evidence-based guidelines and raises ethical concerns regarding equal access to mental health care. This issue is not limited to Germany and the UK–internationally, psychotherapeutic services for schizophrenia remain alarmingly scarce. In China, however, recognition of this problem led to significant improvements in service availability between 2007 and 2013 (Ding et al., 2022).

Psychodynamic approaches appear relatively marginalized across many countries (Yeung, 2021). The limited availability of these treatments further contributes to gaps in psychotherapeutic care, leaving a significant proportion of schizophrenia patients without access to specific psychotherapeutic treatment according to the current guidelines.

Psychodynamic group therapy was introduced at a psychiatric outpatient clinic 25 years ago to address the lack of psychodynamic psychotherapy available to patients with schizophrenia. A “slow open” group therapy model emerged from therapist observations and patient feedback, integrating key psychodynamic principles to enhance therapeutic engagement.

The group is conducted as a continous outpatient group with six to eight participants and meets once weekly for 60 min. It is led by one therapist and one co-therapist, allowing for continuity and the integration of different clinical perspectives. Sessions are oriented toward current themes brought in by group members. Over time, a semi-structured group frame has developed, including a consistent opening and closing sequence, which supports containment and group cohesion. Rather than using directive or frequent interpretative interventions, therapists focus on maintaining the therapeutic frame through structuring, containment, and the regulation of group boundaries, thereby diluting the intensity of dyadic processes (which can easily become overwhelming for those suffering from schizophrenia) and facilitating the negotiation of relational dilemmas within the group (von Haebler, 2015a).

To support quality assurance in clinical practice, our research group began audio recording and additional documentation of group interactions in PDGT sessions at the psychiatric outpatient clinic in 2019, with additional use for research purposes.

The COVID-19 pandemic impacted the group therapy in multiple ways. Within the naturalistic setting, the pandemic offered valuable insights into patients’ perceptions of the crisis. This paper hypothesizes that the pandemic acted as a catalyst, intensifying core psychodynamic dilemmas in individuals with psychosis, particularly proximity-distance dilemmas. As such, the crisis served as a magnifier, making both patient dilemmas and the therapeutic benefits of PDGT more visible.

COVID-19 had a profound impact on patients and their therapeutic care, prompting PDGT and other services to adapt to the conditions of the pandemic (Brown et al., 2020; Šago et al., 2020; Wajda et al., 2022).

Challenges arose due to pandemic-related restrictions and increased crises among participants. Recent qualitative studies explore the perspectives of people with schizophrenia on the pandemic (Karanci et al., 2023; Kotlarska et al., 2021; Lyons et al., 2021).

Conspiracy theories, apocalyptic fears, and isolation dilemmas affect society at large (Haas, 2020), but for individuals with schizophrenia, they evoke core, symptom-producing dynamics that are present even beyond the pandemic context (Mentzos, 1997).

This study explores which themes emerged in PDGT sessions with patients diagnosed with schizophrenia or schizoaffective disorder during the initial outbreak of the COVID-19 pandemic, as identified through qualitative content analysis. The main themes that were central to patients during the early phase of the pandemic, as identified through qualitative content analysis, are subsequently compared to Mentzos’ theory of psychosis to assess conceptual alignment.

This study aims to identify core dilemmas in schizophrenia and schizoaffective disorders, providing deeper insight into the mechanisms of PDGT. An understanding of psychosis based on psychodynamic theory demonstrates the approach and potential of PDGT. In this way, this study shows how PDGT works precisely by bringing the specific dilemmas of people with schizophrenia into the group making it accessible for psychotherapeutic interventions. By sharing these insights, we aim to encourage psychotherapists to engage with schizophrenia patients and advocate for stronger integration of PDGT in mental health systems.

The second aim of this study is to demonstrate how challenging conditions in group therapy, such as changes in structure, telephone sessions, and the impact of global crises on participants, lead to the destabilization of the group and, in turn, the patients. The findings highlight the need for therapeutic services to prioritize continuity of group therapy during times of global crises, such as climate change, geopolitical conflicts, and pandemics, which increase the demand for resilient and effective mental health support (Chmielewski, 2023). This study is intended to contribute to high-quality, guideline-oriented care for people with psychoses, particularly in times of social upheaval.

In addition, qualitative content analysis according to Mayring (2015) is applied to audio recordings of group sessions, offering a direct exploration of the therapeutic processes. This approach moves beyond interviews or discourse analysis (Karatza and Avdi, 2011), providing valuable real-time insights into group dynamics.

## 2 Materials and methods

### 2.1 Ethical standards and funding

The study received ethics approval from Charité Universitätsmedizin Berlin ethics committee, and participants provided written informed consent. They were informed that they could withdraw their consent at any time. The transcripts were anonymized to safeguard participants’ identities. The datasets generated for this study are stored on secure servers at Charité – Universitätsmedizin Berlin. Anonymized data can be made available by the corresponding author upon reasonable request, in line with institutional and ethical guidelines. The authors declare that the research was conducted in the absence of any commercial or financial relationships that could be construed as a potential conflict of interest.

This research was partially supported by an institutional starting grant from the International Psychoanalytic University (IPU), Berlin. The study was retrospectively registered in the German Register of Clinical Trials DRKS00033756 on 20/03/2024 due to unforeseen administrative delays.

### 2.2 Research design and data collection

The data were collected from the PDGT for patients with schizophrenia spectrum disorders as described above. Therapy sessions were documented by an intern present from February 2019 to August 2020 and recorded using tape recordings.

Audio recordings were used for analysis, as video recordings were ethically inappropriate and could disrupt the natural group process. While non-verbal cues like facial expressions and gestures are relevant, especially for patients with schizophrenia, a camera might have influenced participant behavior. Choosing audio recordings minimized observer bias but limited non-verbal communication analysis.

This study focuses on sessions conducted between March 2020 and June 2020, covering the initial lockdown phase. This includes the period of first and most severe curfews, closed stores, and restaurants, i.e., the period of the so-called first lockdown [phase 1 according to Schilling et al. (2021)]. Six sessions from early April to mid-May were conducted via telephone.

The transcripts were retrospectively transcribed and analyzed by a doctoral student trained in qualitative content analysis who did not participate in the therapy sessions. The research team included a psychiatrist with clinical experience in group psychotherapy and qualitative research, a doctoral candidate in psychology and psychotherapy research, and the psychiatrist who facilitated the group therapy. The results were discussed and refined in research workshops.

### 2.3 Study group

The inclusion and exclusion criteria of the study corresponded to those of the group therapy due to the retrospective data evaluation. Participants (*n* = 7) had a diagnosis of schizophrenia [F20.0 (85.7%) or F20.1 (14.28%)]. Exclusion criteria included a prominent addictive disorder requiring treatment. Additional secondary diagnoses included adjustment disorder F43.2 (16.67%), generalized anxiety disorder F41.01 (16.67%), and a history of substance use disorder F19.20 (16.67%).

**FIGURE 1:**
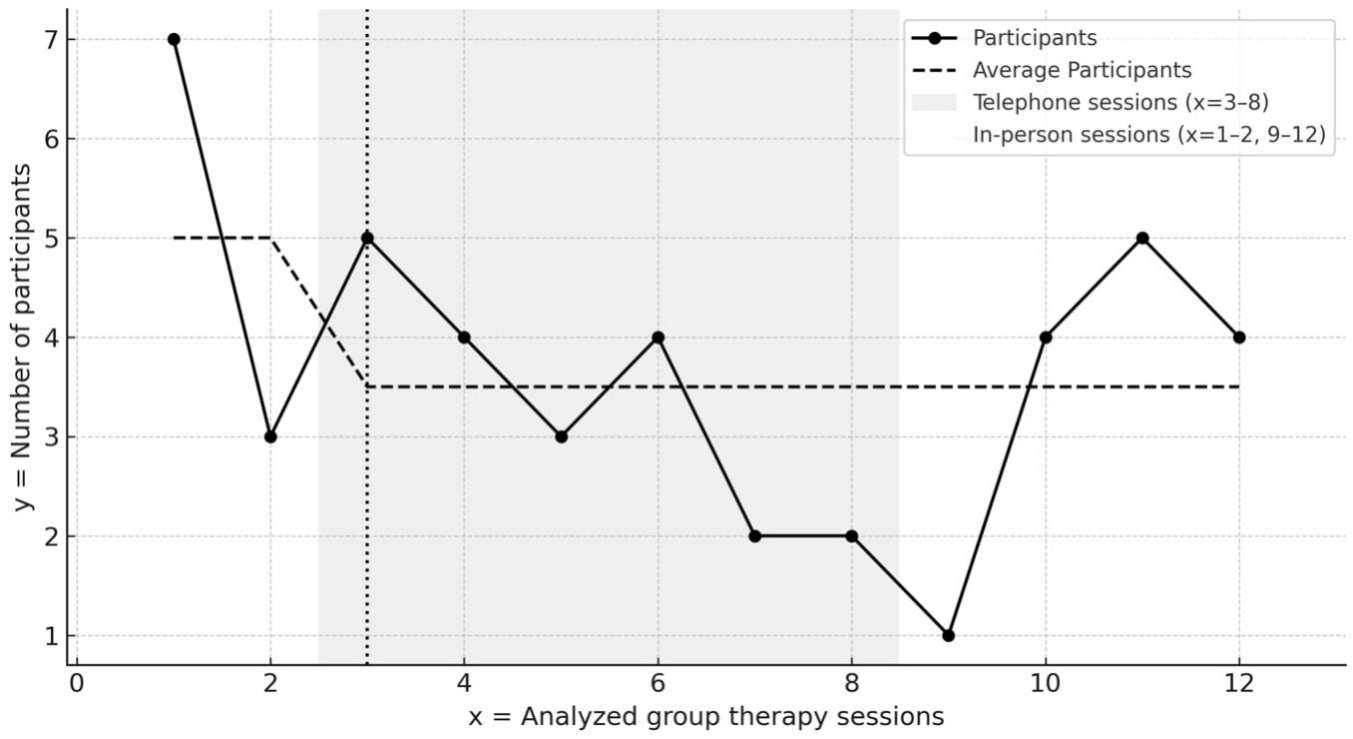
Participant numbers during the observed period (March–June 2020). Three therapy sessions were canceled between *x* = 2 and *x* = 3 due to pandemic regulations. A decline in participant numbers can be seen throughout the analyzed period. Sessions at *x* = 1–2 were conducted in person, while sessions at *x* = 3–8 took place via telephone.

The number of participants per session varied from seven to one during the pandemic period (see Figure 1). Three sessions were canceled due to pandemic restrictions, and a decline in participant numbers was observed throughout the analyzed period.

#### 2.3.1 Data preparation and analytical approach

Group psychotherapy sessions were transcribed verbatim; non-verbal aspects such as pauses (marked with a bullet point every 3 s), laughter, and filler words were also noted. A semicolon indicates a shift in the direction of thought. When translating from German into English, participants’ statements were translated as closely as possible to the original, with efforts made to also convey the underlying meaning in cultural idioms and metaphors. The identity of the participants was protected by the use of pseudonyms.

The transcripts were analyzed with the computer program MaxQDA (VERBI Software, 2022) according to Mayring’s summarizing qualitative content analysis (Mayring, 2015).

This method was chosen because it allows for the structured processing of large text volumes while maintaining methodological rigor.

Each session is labeled by its respective date (e.g., 10.03.2020). Within each transcript, every individual utterance or segment is numbered automatically by the software and referred to as an item (e.g., item 53). Citations in the text, therefore, follow the format (date, item number), such as (10.03.2020, item 53), indicating the source session and the corresponding line number in the transcript.

### 2.4 Category formation

In the following analysis, a distinction is made between “topics,” “themes,” and “categories.” Topics refer to the specific contents raised by group members during therapy sessions. They serve as the starting point for the analysis. Themes represent overarching patterns of meaning that emerge from the interpretative engagement with the topics. Categories constitute the analytical framework used in the qualitative content analysis, according to Mayring, and were developed inductively during the coding process.

**FIGURE 2:**
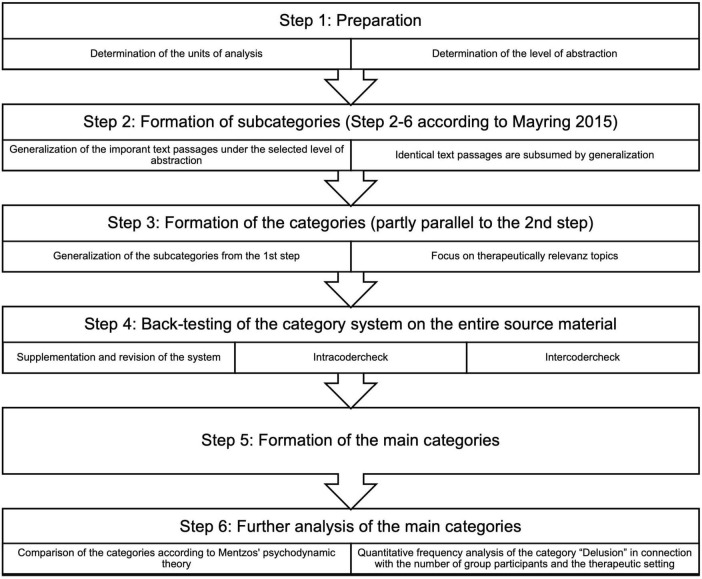
A process model of the summary content analysis of group psychotherapy sessions, adapted from Mayring (2015).

As a level of abstraction, we did not choose paraphrases but statements that closely resembled what was said, yet were abstract enough that other group participants could have shared them (see Figure 2, Step 1).

While the study aims to draw conclusions about group psychotherapy, the research question focuses exclusively on participants’ subjective experiences within that group psychotherapeutic setting. Accordingly, only participants’ statements were paraphrased, ensuring a consistent and comparable level of analysis across all coded units. Therapeutic interventions were not coded, as this would have required a process-analytic approach and shifted the focus away from a content-descriptive analysis; they are therefore conceptualized as part of the enabling framework and reflected indirectly in the emerging themes.

The doctoral student analyzed each session in detail, forming initial categories or assigning text passages to existing ones. Inductive category formation was applied, with subcategories emerging for similar topics, delineating dilemmas or specific life areas from a psychodynamic perspective (see Figure 2, Steps 2–3).

An example of category formation is shown with the following quote:

“And I would rather be infected than go into quarantine without him” (03.03.20 item 102).

The statements as close as possible to what was said were “Fear of Being Alone in Quarantine/Infection” (*n* = 4). It was then classified under the subcategory “Emotional Self-Sufficiency” (*n* = 103), which is part of the category “Autonomy and Dependence” (*n* = 131), and the main category “care and self-care” *n* = 131.

After coding all twelve sessions, the categories were reviewed holistically to refine overlaps and ensure consistency. The final category system consisted of 15 categories, which were further abstracted into four main categories (see Figure 2, Steps 4–5).

**FIGURE 3:**
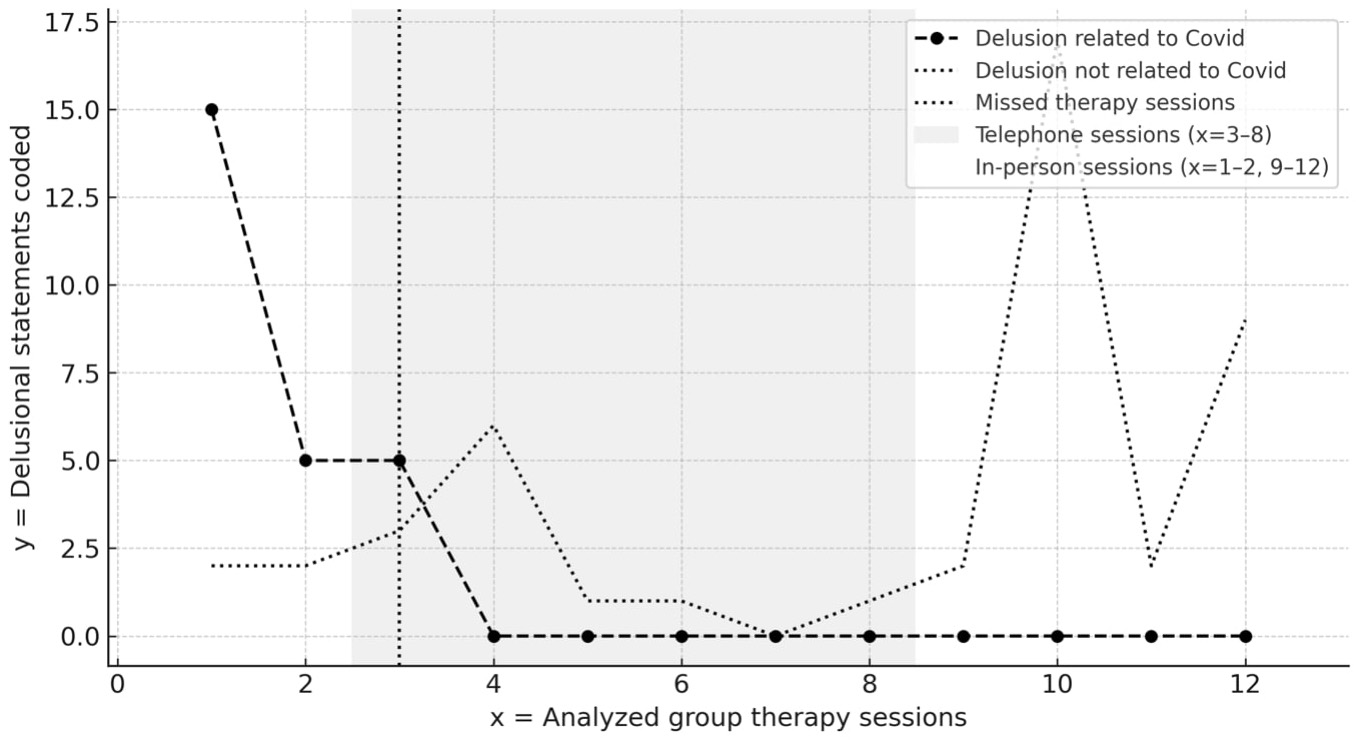
Delusional statements in group sessions: COVID-related vs. non–COVID-related. In-person sessions took place during phases *x* = 1–2 and *x* = 9–12, while telephone sessions were conducted during phases *x* = 3–8.

As a next step, selected category frequencies were analyzed, partly about other factors (Figure 3).

*The core themes were then compared retrospectively with Mentzos’ psychodynamic theory of psychosis to provide a clinical framework for understanding the emerging patterns. Importantly, the categories had been developed inductively and were not shaped by the theory in advance.*

### 2.5 Deviations from the process model by Mayring

In the process model of summarizing content analysis (Mayring, 2015), it is mentioned that in the case of large amounts, paraphrasing, generalization, and reductions can take place in one step (see Mayring, 2015, p. 70). Due to the combination of these steps, the procedure strongly resembles inductive category formation (see Mayring, 2015, pp. 85 “inductive category formation”; see Figure 2, Step 2).

Summarizing ensures that entire sessions are considered, while the condensed steps allow for the analysis of large datasets. This not only enables the examination of individual sessions but also provides insights into overall developments.

### 2.6 Quality criteria

All coding was done manually to maintain accuracy and contextual sensitivity. MAXQDA (VERBI Software, 2022) was used to structure and trace the coding process, ensuring transparency.

The intracoder reliability was confirmed by a complete re-evaluation of the original material after category formation. Intercoder reliability was ensured by independent categorization of 50% of the sessions by a psychiatrist from the research team, followed by discussions of discrepancies. Given the interpretative nature of psychodynamic analysis, differences in categorization were often attributed to countertransference phenomena, which were resolved through team discussions rather than relying on percentage-based reliability calculations (see Figure 2, Step 4).

To ensure scientific rigor, qualitative content analysis adhered to established quality criteria. Category formation followed systematic summarization and inductive structuring principles, with all categories traceable to the original transcripts.

## 3 Results

The themes discussed in the group psychotherapy during the outbreak, as identified through qualitative content analysis, are presented below in order of category frequency.

### 3.1 Loss of something meaningful

The participants talked most often about topics in the category “loss of something meaningful” (*n* = 245). In addition to discussing death and the value of life (coded as 1.1. in Table 1), there was also frequent mention of problems in the group and what the group means to the participants (coded as 1.2. in Table 1). Vulnerabilities and experiences of loss due to the COVID-19 pandemic were also discussed (coded as 1.3. in Table 1).

**TABLE 1:** Overview of the category system with main categories, categories, and subcategories.

Main category with category	Examples from subcategories (cut off *n* = 3, max. 5)
**1. Loss of something meaningful (*n* = 223)**	
1.1. Life and death (*n* = 41)	1.1.1. Reflection on life 1.1.2. Acceptance/trust 1.1.3. Fear of death
1.2. Group (*n* = 156)	1.2.1. Problems in the group 1.2.2. Meaning, value, and function of the group
1.3. COVID-19 (focus: loss and death) (*n* = 22)	1.3.1. Support on the outside is missing because of Corona 1.3.2. Loss of day structure threatening 1.3.3. Fear of losing relatives
**2. Paranoia (*n* = 158)**	
2.1. COVID-19 (focus: theories) (*n* = 18)	2.1.1. Alternative/conspiratorial theories 2.1.2. Current situation is threatening 2.1.3. Danger is less bad than conveyed
2.2. Trust/fear (*n* = 55)	2.2.1. Distrust/influence in the group 2.2.2. Distrust of media/authorities/discourse 2.2.3. Distrust of other people 2.2.4. Role models
2.3. External world/political situation (*n* = 45)	2.3.1. World outside is evil/threatening/suffering 2.3.2. Outside fraudulent/delusional 2.3.3. External puzzles or disorientation
2.4. Psychiatry (*n* = 40)	2.4.1. Lawsuits 2.4.2. Justified aggression 2.4.3. Fraud in psychiatry 2.4.4. Unjustified violence/coercion
**3. Care and self-care (*n* = 131)**	
3.1. Autonomy and dependence (*n* = 103)	3.1.1. Emotional self-sufficiency 3.1.2. Dependence on an ambivalent/idealized person 3.1.3. Material self-sufficiency
3.2. Money/buy (*n* = 19)	3.2.1. Toilet paper crisis 3.2.2. Financial compensation
3.3. State/society/individual (*n* = 9)	3.3.1. Individual dependence on society
**4. Proximity and distance (*n* = 102)**	
4.1. Distance and demarcation (*n* = 70)	4.1.1. Separation of feelings/mood/opinions of others 4.1.2. Emotional distancing 4.1.3. Preservation through isolation
4.2. Proximity and symbiosis (*n* = 32)	4.2.1. Loss of self in close relationships 4.2.2. Blurred emotional boundaries 4.2.3. Inseparable expectations

The main categories (e.g., 1.) are listed on the left, each followed by their respective categories (e.g., 1.2.) and frequencies (e.g., *n* = 41). To avoid visual overload, only subcategories (e.g., 1.2.1.) that were mentioned at least three times are displayed, with a maximum of five per category. Selection was based on their relevance to the overarching themes of the main categories.

In the first sessions, triggered by the news about the coronavirus, the participants reflected on their own lives.

Burkhard: “It is; you are reminded of death by the fact that death now seems more real than before, that if you now think about, if I now get the virus and die from it, how did I live? What would I have liked to have done better, or where do I still have (.).” (10.03.2020, item 53)

Many participants were burdened by the loss of daily structure. Some reported feelings of paralysis or depression:

Mina: “Yeah, I know from me it’s kind of depression (…) at this time, with no structure, no depression med, no; so going through everything all by myself and stuff. I know it’s also a kind of depression.” (21.04.2023, item 99)

Some participants were also worried about the loss of their relatives.

Michael: “But the problem for me is: I have a daughter, so I’m really worried, and if they should really lock down cities or something, huh;” (03.03.2020, item 99)

Tobias: “For example, I have my mother, who is missing a piece of her lung because she had cancer and she underwent surgery a long time ago, but she also has asthma (.). If she got that (Editor’s note: COVID-19), she might not survive, so that worries me too.” (07.04.2020, item 140)

Group psychotherapy itself was the topic of much discussion and reflection at the beginning and end of the evaluation period (*n* = 156) (coded as 1.2. in Table 1).

There was a disintegration of the group during the telephone sessions, which was expressed by the low number of participants. One participant complained that the common thread was missing. The loss of group members - understood as their absence from sessions - was also discussed:

Julia: “Well, I have the feeling that Carolin and Burkhard are somehow missing, and that if they were there, it might be better again. - But that the themes don’t seem connected, because in the past it was like that, that all therapy sessions, there was such a coherent theme, they all built on each other and developed further in one direction and now it is like that, that the connection to the previous session is somehow missing.” (09.06.2020, item 55)

Another participant repeatedly announced that she would leave the group because of existing problems in the group (coded in the subcategory 1.2.1. “Problems in the group” in Table 1).

At the same time, the same participant talked about how she cared about the group:

Viktoria: “Mhh (thoughtful) – yes, so a bit wistful, so I also want the group to do well and I also want to contribute and actually be a part that cushions something when someone is not doing well.” (09.06.2020, item 78)

The group seemed to have a high value for many participants, which became apparent especially during the time of group disintegration (coded as 1.2.2. in Table 1).

### 3.2 Paranoia

In the sessions, participants frequently made contributions (*n* = 158) in which they expressed distrust, for example, of the public discourse about COVID-19 or of other reference persons, group participants, or general political events (coded as 2.2. in Table 1). The description of one participant after his stay in a psychiatric hospital was also special in its delusional character (coded as 2.4. in Table 1). The statements were similar in that the basic tone was threatening, and something bad was suspected on the outside.

Alternative and conspiracy theories about the coronavirus were discussed, especially during the outbreak period (coded as 2.1. in Table 1). The news was experienced as overwhelming and questionable. All 18 delusional statements about COVID-19 were made in the first three sessions, and their frequency decreased with each session (see Figure 3). Most of the theories were not constantly present with delusional intensity but did arise frequently in different guises.

Burkhard: “Well, I said it before that I; that I believe that this emergence of the coronavirus, that this is not true, but I believe that it was produced in a bioweapons laboratory and then released on the market.” (03.03.2020, item 74)

Moreover, in the sessions, the world in general was experienced as evil, threatening, and suffering.

Mina: “(…) I don’t like (…) all this competition and procedure and here and some have to emigrate everywhere, and the others have to beat them to death everywhere at the borders and so on. So when I see these pictures, terrible, yes, frightening, terrible (coughs).” (03.03.2020, item108)

Some posts had delusional components in addition to the conspiracy theory content:

Mina: “To be honest, I also feel very pressured, because there’s a Chinese embassy at work on the way to the S-Bahn and there are demonstrations across the street every day about issues in China, oppression, and so on, organ theft, and so on, and now also Corona. And I feel so pressured every time. When I get there, they look at me with such eyes and as if they wanted to take away everything I still have inside me. As if I had to donate all the drops of blood that were still flowing in me, somehow, and I have to do it immediately. And now I realize that China now suddenly has such a relevance in the news (.).” (03.03.2020, item 73)

It was noticeable that the contributions were initially not rigid and that the topics of one participant extended over a maximum of two sessions (cf. 03.03.2020, item 73 and 10.03.2020, item 29).

In this category, delusions, which were more consistent and conspicuous, appeared only later in the course.

One participant was convinced that her environment wanted to harm her by keeping her “fat.”

Mina: “(.) I somehow have the feeling that being fat is promoted more, and being thin is not so promoted. I have a few reasons. First, my (Editor’s note: Name of her antidepressant) was discontinued (.), and I can’t go there (to rehab) now either, because this structure also makes me thin again, and if I stay at home, that also brings me more weight. (.) all my girlfriends and so on, they are also always against me having a boyfriend, they want to talk me out of it and when I tell them about someone, “Oh no, what kind of guy is he,” “Break up” and so on, “What kind of relationship is that” and this not ending life without having a relationship, that also makes me fat and so on (.).” (21.04.2020, item 71)

Another participant’s delusion included many areas, such as his father, his social environment, and his involuntary psychiatric admission, which was prompted by his acute stranger-threatening behavior. He described that the psychiatric unit deliberately colluded with the police to provoke him into a fit of rage and to forcibly house him immediately afterward.

Michael: “And the funny thing was, they waited for it to happen, because after I threw the TV in, exactly 3 s later, the policemen were standing next to it and arrested me; what do you call it; fixed, handcuffed, then on the couch and so on. They waited for it specially – on purpose.” (02.06.2020, item 117)

The anger he had toward his family and his psychiatric hospitalization became the central topic of the last sessions.

### 3.3 Care and self-care

The topics around care were frequently discussed by the participants, also in connection with the pandemic (*n* = 131). This main category included statements about autonomy and dependence, money and buying, and the participants’ relationship to society and its organizational structure (coded 3.1–3.3. in Table 1). A recurring question was whether the participants were able to take care of themselves materially or emotionally (coded as 3.1.1. and 3.1.3. in Table 1).

One of the participants took care of himself materially by “prepping,” i.e., stocking up on food for a long period (coded as 3.1.3. in Table 1). In this way, he avoided the feeling of being dependent on society due to the COVID-19 crisis (coded as 3.3.1. in Table 1).

Burkhard: “So what is actually good is to find an active way of dealing with it. I have now read my prepping and (…) crisis forecast books again and on Tuesday I was on the road, I got 2.5 kilos of powdered milk, long shelf life, with which you can then make over 10 liters of milk and I also always make sure that I have canned goods, meat, which can be kept for years or sugar or that I also get coffee or tea as a supply (.). But even if you were to get Corona, you could say that you have done everything to be as prepared as possible, so to speak (.).” (07.04.20 item 153)

He also said prepping provided him with emotional security and a way to deal with the outbreak of the pandemic.

In contrast to this participant who is helping himself to autonomy, another participant is worried by the idea of infection (07.04.2020, item 139) and hopes not to be alone in quarantine.

Viktoria: “(.) So if I were in quarantine, (.) because I live with my husband, and if it wasn’t so bad with me or with him, then you could just cure it at home, (.) and somehow get through it together. If we both have it. And I would rather be infected than go into quarantine without him (laughs), and I think, I hope that he also sees it that way somehow, so I haven’t talked to him about it yet.” (03.03.20 item 102)

### 3.4 Closeness and distance in relationships with others

In the relationships in the group and the outside world, the participants described, in addition to their care dilemma, dilemmas in the area of family, partnership, housing, work, and also in the group, in which it was often (*n* = 112) about separating oneself mentally. The participants found it difficult to distance themselves from the feelings, moods, or opinions of others. When they engaged with the other, their sense of their “own” was lost (coded as 4.2.1. and 4.2.2. in Table 1). While work does provide a sense of security in terms of daily structure and thus self-care, it is also an area where participants seem to find it difficult to set boundaries (*n* = 29).

Distancing oneself from the expectations of others was a recurring theme (coded as 4.2.3. in Table 1). One participant, for example, repeatedly described how her friends consumed her time and attention. After another such encounter, she reported having forgotten everything else. When the therapist asked whether she remembered the previous session, she replied:

Mina: “No. (laughs) I was invited on the weekend, when my acquaintance babbles all over me, I mean has a lot to tell me, then I forget everything.” (10.03.2020, item 8)

Another participant reported difficulty separating herself from the problems of her father and his partner.

Viktoria: “He (Editor’s note: the father) doesn’t cry that often, he rarely cries, but when it was with my mother, I saw him crying for the first time 5 years ago and then very, very often even, yes, and I just think somehow that he doesn’t want to show that to me, maybe out of protection, because he wants to protect me, but maybe also because he doesn’t want to and can’t bear it himself, and in that situation, if I remember, I got such an extreme feeling, and then I also had the feeling that I had to turn away, because that was while I was working still, so I still had a week of school afterward, and I have the feeling that I can’t go into it like that now, so in retrospect, in the situation in which he told me that, or wanted to tell me, I had the feeling, but I don’t know exactly (…); so I do not remember the situation at all exactly.” (14.04.2020, item 135)

She also identified with her father’s mood to such an extent that she “had to turn away,” otherwise she would not have been able to go about her daily life. Subsequently, she could no longer differentiate between her feelings while explaining and her feelings during the event she was describing. Her memory was lost to her.

### 3.5 Mentzos’ psychosis theory and the dilemmatic constellations

The psychiatrist and psychoanalyst Mentzos (1997) challenged the prevailing view that ego weakness is central in psychoses, as opposed to neurotic disorders, which were more commonly associated with internal conflicts. Based on his clinical experience, he argued that ambivalence - an inner inconsistency - also plays a prominent role in psychotic patients and arises from an inability to resolve dilemmatic constellations satisfactorily. He reinterpreted what had previously been seen as an “ego function disorder” as “psychotic defense, protection, and compensation mechanisms” (Mentzos, 1997, p. 344), offering a new perspective on the psychopathology of psychotic disorders.

“Normal bipolarities” could not be “constructively resolved” (Mentzos, 1997, p. 345) and thus lead to the symptoms of schizophrenia. These polarities span between a self pole and an object pole [see Figure 2 in Mentzos (1997), p. 347]. The figure is titled “Relational and ego-functional aspect of defense modes.” In schizophrenia, the pathognomonic polarities are self-identity and fusion, whereas in affective psychoses, the dilemma of self-worth and object-worth is central (Mentzos, 1997, p. 347). In schizophrenia, the pathognomonic polarities are self-identity and fusion, whereas in affective psychoses, the dilemma of self-worth and object-worth is central (p. 347). Dilemmatic constellations have already earlier been described as pathognomonic for the psychoses in literature by Burnham, Balint, Mahler, Recamier, and Benedetti (Lempa et al., 2017).

The topics “closeness and distance” and “care and self-care” show that dilemmatic constellations seemed to occur again and again in the psychological experience of the participants, which makes a “dialectical and constructive suspension of the opposition” (Mentzos, 1997, p. 345) impossible. The ambivalent relationship between the individual and society (as coded as 3.3. in Table 1) can also be understood as such a dilemma. In these inductively derived main categories, we see clear overlaps with Mentzos’ theory of psychosis.

Following Mentzos’ approach, we operationalized this theory by identifying categories in which fundamentally opposing needs, such as autonomy vs. dependence and closeness vs. distance, are negotiated but remain unresolved. These dilemmas specifically align with Mentzos’ described polarities and were particularly pronounced in the context of the pandemic. In the period observed in this project, the COVID-19 outbreak provided the framework in which dilemmatic responses were made.

In the following, we present two concise examples of representative dilemmatic experiences, while emphasizing that pronounced self–object polarities were observed in many other sessions and thematic categories as well. The dilemmatic constellations can be seen, for example in the fear of loneliness in quarantine on the one hand (coded as 3.1.1. in Table 1) and in “prepping” as an act of counter dependency (coded as 3.1.3. and 3.3.1. in Table 1).

Patient Viktoria described a desire to be infected by her husband rather than be alone in the event of an infection. The situation of being alone at home seemed to represent a threatening, unbearable scenario for her. This group participant defended the dilemma in terms of a “one-sided solution” (Mentzos, 1997). In her case, the “fusion or submission to the object” (Mentzos, 1997, p. 344) meant the - in her mind - preferred infection with the (at this time still in many cases deadly threatening) virus to be able to be close to her partner.

For the participant Burkhard, the situation was not only about the question of isolation and quarantine, but also about being able to take care of himself physically and materially. He fended off the dilemma with the other pole: the solution described by Mentzos as “narcissistic withdrawal” (Mentzos, 1997, p. 433) or “autism” (Mentzos, 1997, p. 345) meant in Burkhard’s case to arm oneself with food purchases in such a way that the isolation could be survived. Withdrawal, compartmentalization, and autism (Lempa et al., 2017; Mentzos, 1997) are the only ways to maintain one’s identity, at the cost of a complete lack of relationships. This same participant also left the group after problems participating in the group therapy via phone and fear of infection. This withdrawal from the group can thus be discussed as a solution to the dilemma. In withdrawing from the group, Burkhard seems to have chosen autistic avoidance over closeness.

## 4 Discussion

This study examined the themes discussed in PDGT for patients with schizophrenia and schizoaffective disorders during the COVID-19 pandemic (results summarized in Table 1), comparing them to Mentzos’ psychosis theory.

The core themes - “loss,” “paranoia,” “(self-)care,” and “proximity and distance” - were central to patients during this period (Table 1). The analysis revealed specific dilemmas, as well as the impact of pandemic-induced changes on group therapy dynamics.

### 4.1 Loss, group disintegration, and delusion

During telephone sessions, the number of active participants significantly decreased (from an average of five to approximately three), struggling to recover even after in-person sessions resumed (see Figure 1). Patients perceived this lack of continuity as distressing, exacerbating feelings of personal loss. These experiences extended beyond therapy, affecting daily routines and concerns about family members (see the coded subcategories in 1.3. in Table 1).

The psychoanalyst Bion originally conceptualized “containment” as a maternal function, in which the mother absorbs the child’s “projected feelings, needs or unwanted parts,” modifying them and then returning them in a bearable way (Stiers, 1995, p. 132). This concept has been extended to group therapy, where the group provides a similar function by holding and reflecting emotional experiences. The shift to telephone sessions and pandemic-related uncertainties disrupted this process, likely contributing to increased delusional symptoms.

The group’s destabilization during phone therapy can be seen as a weakening of its containment function in Bion’s sense. In face-to-face sessions, the group provided a stabilizing frame for psychotic fears, but this function diminished without direct interaction. This may explain the increase in paranoid content, as individual fears were no longer processed collectively.

This study provides a clear indication of a link between continuity of group therapy and patients’ delusional symptoms (Figure 3). Initially, group discussions facilitated the articulation of conspiracy theories and anxieties, allowing for psychodynamic processing. During telephone sessions and the decline in the number of participants, the formation of rigid delusions unrelated to the pandemic likely represented a return to earlier, less flexible attempts at restitution (Freud, 2018) or from Mentzos’ perspective a self-preservatory act of withdrawal.

Group cohesion, as described by Yalom (2015), refers to the sense of belonging and emotional connection among members, which fosters engagement and therapeutic progress. In our study, participants emphasized the value of the group, often in the context of its threatened disintegration, highlighting the protective function of what had been a cohesive therapeutic environment.

Relevant statements can be found in the subcategory “Importance, value and function of the group” (1.2.2.) in Table 1. The importance of the group for the participants and the positive experiences in this context also align with the findings of Schwarz and Matussek (1990). Here, the experience of togetherness–often feared in other contexts–was perceived positively. These results suggest that, when tailored to the specific needs of individuals with schizophrenia, group therapy provides a valuable opportunity to foster meaningful relationships without evoking feelings of threat.

Studies highlight the importance of group cohesion in schizophrenia therapy. Lecomte et al. (2015) found that symptom improvement and self-esteem in early psychosis patients were strongly linked to group cohesion and therapeutic alliance. Rice et al. (2018) showed that structured, long-term group therapy fosters social connections, reducing hospitalizations and supporting personal recovery. The return to “autistic” and socially withdrawn modes of operating was therefore also likely due to the loss of group cohesion, which had allowed non-“autistic” ways of being in the world (Mentzos, 1997, p. 334).

### 4.2 Mentzos’ theory of dilemmatic constellations in group therapy

Our findings support Mentzos’ theory of dilemmatic constellations, particularly regarding the poles of autonomy vs. fusion and care vs. self-care. Two patient examples - one prioritizing symbiotic closeness, the other choosing defensive withdrawal - illustrate these tensions. PDGT appears to offer a setting to process such dilemmas with reduced risk of symptom formation, as it allows relational exploration in a contained, less threatening environment (Štrkalj Ivezić and Urlić, 2015).

### 4.3 Implications for therapeutic practice

Our study underscores the need for continuous group therapy, especially for patients with schizophrenia, who rely heavily on stable interpersonal frameworks.

Given recurring crises, maintaining in-person group therapy should remain a priority to ensure stability for schizophrenia patients. We see signs that the healthcare system will cut back more quickly on the treatment of psychiatric illnesses than on somatic illnesses, even though the need for treatment can be just as important in terms of mortality and expenditure as a physical illness (Barcella et al., 2021; de Mooij et al., 2019). An overreliance on digital formats may be particularly problematic for psychotic patients, who often struggle with abstract or mediated communication. The potential of hybrid models combining in-person sessions with digital elements requires further evaluation, particularly regarding their suitability for psychotic patients.

Further research should investigate the impact of discontinuity and changes in psychotherapeutic services for patients with schizophrenia on an international scale. In Germany, the number of cases in individual psychotherapy recovered much more slowly than in group psychotherapy after the pandemic (Deutsche Psychotherapeuten Vereinigung e. V., 2021). In Australia, evidence suggests that the effectiveness of psychosis treatment declined during the pandemic, highlighting potential limitations of telemedicine (Cotter et al., 2024).

Psychotherapeutic care for schizophrenia patients must not fall below already insufficient pre-pandemic levels, regardless of the crisis type (e.g., economic downturns, geopolitical instability).

Within therapy itself, key aspects should include a strong focus on continuity (Jesser et al., 2021), consideration of individual coping strategies (e.g., isolation vs. fusion), and examining cohesion-strengthening interventions as a therapeutic tool. Additionally, greater emphasis should be placed on understanding individual crisis processing within the framework of dilemmatic constellations, ensuring that therapeutic approaches acknowledge and address the specific ways patients navigate crises.

Although rooted in the German psychodynamic tradition, Mentzos’ concept of dilemmatic constellations provides a valuable framework for understanding psychotic dynamics internationally. In countries where cognitive-behavioral approaches dominate, incorporating psychodynamic theories could enrich schizophrenia treatment approaches (Stuke et al., 2020).

Psychodynamic models could complement existing approaches, such as Open Dialogue in Finland (Cooper et al., 2020) or culturally sensitive therapies in low- and middle-income countries (Asher et al., 2018), by offering a deeper understanding of the psychodynamics of psychotic processes, an understanding that can exist alongside other explanatory frameworks. This study highlights the potential in using a psychodynamic approach, especially as related to group processes, to understand and treat psychotic illnesses.

Although therapeutic interventions were not directly analyzed, the findings allow for theory-informed implications regarding how such a group needs to be facilitated. In line with manuals of psychodynamic psychotherapy for schizophrenia (e.g., Lempa et al., 2017), the therapist’s primary task is not interpretative work but the maintenance of a stable, containing group frame that can tolerate intense affect and ambiguity. Central dilemmas such as closeness and distance therefore emerge mainly within the relationships between group members and are mitigated in the here and now by the presence of other affected group members and a therapist who facilitates the group process through a holding non-intrusive stance. This differs significantly from a dilemma emerging in a dyadic relationship to a therapist.

Typical interventions focus on supporting group cohesion, regulating boundaries, and bringing emotionally charged material back to the group, thereby enabling psychotic dilemmas to be verbalized and then often negotiated with the help of the group.

### 4.4 Limitations

Mayring’s qualitative content analysis is a content-descriptive method that does not account for group communication structures (Mörtl and Gelo, 2014). As a result, non-verbal cues and unspoken dynamics, which are crucial in psychodynamic therapy, could not be analyzed. However, this study compensates by providing in-depth thematic insights that are highly relevant to the treatment of patients with schizophrenia.

The exclusive use of audio recordings further limits the assessment of non-verbal communication. Although video recordings might have enriched the data, they could have influenced group interactions and distorted results. Future studies could explore alternative methods such as discreet video analysis or qualitative observational techniques to better capture non-verbal processes, though the current approach ensured unobtrusive data collection and naturalistic group dynamics.

A key limitation is the lack of pre-pandemic comparison data. Nevertheless, the inductively derived core categories align with psychodynamic theory and provide valuable insights into schizophrenia-specific patterns, particularly regarding proximity-distance dilemmas.

One might argue that focusing exclusively on participants’ statements, without coding therapists’ interventions, limits the ability to directly attribute the observed processes to specific therapeutic actions. However, the aim of the study was not to analyze intervention techniques but to examine which themes and dilemmatic constellations become observable within a psychodynamically facilitated group setting. Unlike interview-based designs, which rely on participants’ retrospective self-assessment and explicit reflection on their experiences, the PDGT group context enables the direct observation of how themes emerge, are negotiated, and shape relationships over time, including ruptures, withdrawal, and changes in participation. Future research could therefore build on the present findings by systematically examining therapeutic interventions and their influence on group processes and participants’ experiences.

It remains unclear whether the increase in delusional symptoms resulted from pandemic-related stressors, group disintegration, or individual disease progression. Longitudinal studies could help clarify this, though feasibility in naturalistic settings is limited. Since a control group was not feasible, distinguishing pandemic-related effects from general disease progression remains challenging.

It is also uncertain which dilemmas would have emerged independent of the pandemic. However, some themes, such as prepping as self-protection versus fusion with a partner, were explicitly linked to COVID-19. A control group of non-schizophrenic participants could help differentiate pandemic-related challenges from disorder-specific dilemmas, though this was also difficult to implement in the naturalistic setting.

The increase in rigid, pandemic-unrelated delusions correlated with the decline in group participation, suggesting group disintegration as a potential contributing factor.

However, topic frequency changes should not be prematurely interpreted as symptom progression, as group dynamics influence which topics are amplified or diminished (Figure 3). While this correlation remains unclear, the findings offer clinically relevant insights into the impact of therapy disruptions on psychotic processes.

The study relies on retrospectively transcribed group sessions, making *post hoc* interpretation biases possible. However, this was minimized by first developing categories in a data-driven manner, followed by theoretical classification. Additionally, a second coder reviewed the data to reduce preconceived biases, increasing reliability.

A significant limitation is of course the sample size. However, given the dearth of literature on PDGT a small study can be important for directing attention to this under-researched therapy and encouraging further exploration, in diverse settings, using the themes identified. The correlation between the emergent themes and the established literature on the psychodynamics of psychosis offers hope that future research might establish this connection more emphatically, and that a promising psychotherapeutic modality will gain traction internationally.

## 5 Conclusion

This study highlights the stabilizing potential of PDGT for individuals with schizophrenia, particularly during crises. The analysis of group dynamics during the COVID-19 pandemic demonstrates how therapeutic discontinuity may have contributed to a relapse in schizophrenia symptoms in group participants. At the same time, the psychodynamic paradigm offers an explanatory framework by emphasizing the significance of dilemmatic constellations. The findings advocate for prioritizing group therapy in schizophrenia treatment and ensuring its stability even in times of crisis.

To our knowledge, this is the first study using qualitative content analysis to explore audio recordings of group psychotherapy sessions. This study contributes to the nascent research into this unique and potentially transformative treatment.

## Data Availability

The raw data supporting the conclusions of this article will be made available upon reasonable request, subject to ethical and data protection considerations.
